# Immune Landscape of CMV Infection in Cancer Patients: From “Canonical” Diseases Toward Virus-Elicited Oncomodulation

**DOI:** 10.3389/fimmu.2021.730765

**Published:** 2021-09-08

**Authors:** Ranim El Baba, Georges Herbein

**Affiliations:** ^1^Department Pathogens & Inflammation-EPILAB EA4266, University of Franche-Comté UBFC, Besançon, France; ^2^Department of Virology, Centre hospitalier régional universitaire de Besançon (CHRU) Besançon, Besancon, France

**Keywords:** HCMV, immunosuppression, immune evasion, immunosenescense, TME, oncomodulation, therapeutic approaches

## Abstract

Human Cytomegalovirus (HCMV) is an immensely pervasive herpesvirus, persistently infecting high percentages of the world population. Despite the apparent robust host immune responses, HCMV is capable of replicating, evading host defenses, and establishing latency throughout life by developing multiple immune-modulatory strategies. HCMV has coexisted with humans mounting various mechanisms to evade immune cells and effectively win the HCMV-immune system battle mainly through maintaining its viral genome, impairing HLA Class I and II molecule expression, evading from natural killer (NK) cell-mediated cytotoxicity, interfering with cellular signaling, inhibiting apoptosis, escaping complement attack, and stimulating immunosuppressive cytokines (immune tolerance). HCMV expresses several gene products that modulate the host immune response and promote modifications in non-coding RNA and regulatory proteins. These changes are linked to several complications, such as immunosenescence and malignant phenotypes leading to immunosuppressive tumor microenvironment (TME) and oncomodulation. Hence, tumor survival is promoted by affecting cellular proliferation and survival, invasion, immune evasion, immunosuppression, and giving rise to angiogenic factors. Viewing HCMV-induced evasion mechanisms will play a principal role in developing novel adapted therapeutic approaches against HCMV, especially since immunotherapy has revolutionized cancer therapeutic strategies. Since tumors acquire immune evasion strategies, anti-tumor immunity could be prominently triggered by multimodal strategies to induce, on one side, immunogenic tumor apoptosis and to actively oppose the immune suppressive microenvironment, on the other side.

## Background

HCMV or human herpesvirus 5 (HHV-5) has co-evolved with mammalian hosts over millennia infecting almost 83% of the world’s population, impending 100% in developing countries (1). After initial infection, HCMV can establish lifelong persistence within its corresponding host as well as possessing the reactivation potential; viral persistence depends on composite interactions among various viral and host determinants. Such interactions mostly generate an equilibrium between the immunocompetent host and the virus itself. In the host, HCMV infrequently causes symptoms unless this balance is demolished by the minimized host immune proficiency (atypical settings) leading to substantial pathology (1, 2). Upon viewing several forms of viral-host interaction, the explicit HCMV reactivation in immunosuppressed patients (organ transplant recipients) and immunocompromised patients (septic patients, elderly, HIV-infected patients, etc.) is considered a well identifiable disease state (3, 4). Hence, in immunocompetent patients, HCMV is considered a multifaceted beta herpesvirus that is viewed as an asymptomatic and mildly pathogenic virus, but may nevertheless cause chronic infections along with acute and serious complications in immunocompromised individuals (5). HCMV persistence can also have a key influence on the host, even in healthy carriers, through the attenuation of innate and adaptive immune responses since HCMV starts to counteract several host immune response mechanisms required to control the infection (1, 2). HCMV potentially triggers the host immune responses starting by the mechanisms of innate immunity, including inflammatory cytokines resulting from virus/cell binding and NK cell induction which consequently drives adaptive immune responses, involving production of antibodies and the initiation of CD4^+^ and CD8^+^ T-cell responses. However, HCMV encodes various immune evasion mechanisms; hence, expressing several genes that influence both innate and adaptive immunity (5, 6).

HCMV, a leading viral cause of birth defects, has been linked to several mortality and morbidity conditions (7). The stage of HCMV acquisition may affect the range of associated clinical manifestations and the effectiveness of the immune responses exerted against HCMV (7, 8). Regarding congenital CMV infection, neurological defects might be experienced as mental retardation, cerebral palsy, and hearing impairment; newborns may experience either symptomatic or asymptomatic infections (7, 9, 10). Symptomatic infections might cause petechiae, low birth weight, jaundice, hepatosplenomegaly, seizures, and microcephaly; they appear to be more common in newborns infected during the first trimester of pregnancy (7). In case of premature birth, sepsis and respiratory distress can develop (11). Compared to adults, these observations indicate that controlling CMV replication is restricted during early stages of life and is associated with delayed immune responses and increased risk of symptomatic infection (7). Years later, persistent HCMV infection might be considered a potential risk factor exacerbating age-associated diseases and immunosenescence which is defined as the age-associated deterioration in overall immune condition (12–14) although some studies have indicated that the link between HCMV and immune aging is obscure (3, 15). Further, various stimuli can induce HCMV reactivation; it might be triggered in SARS-CoV-2 infected patients (16, 17) thus exacerbating the risk of coronavirus disease 2019 (COVID-19) (18, 19). Even if this interaction is still elusive and additional large scale studies are recommended (16), CMV testing and treatment should be taken into consideration in such critical conditions (18). CMV status must be taken into account for several vaccine responses, especially cancer despite the use of HCMV-based therapeutic cancer vaccines (20), since it has been suggested that with advanced age and due to CMV-associated altered immunity in both healthy and immunocompromised hosts, vaccine immunogenicity was modulated (21–23). Thus, recent studies are concerned about targeting HCMV to decrease the sensitivity to other infectious diseases and cancer, and to prevent poor responses to vaccination (21, 22).

The contribution of HCMV infection in late inflammatory complications highlights its potential association with chronic diseases, such as atherosclerosis, chronic rejection following solid-organ transplantation, and malignancies (24). Recent investigations have reported the prevalence of HCMV infection in tumoral tissues of malignancies such as malignant glioma, breast and colon cancer, negative Hodgkin’s disease, Epstein-Barr virus (EBV), liver cancer, cervical cancer, and prostatic carcinoma (1, 25, 26). Despite the fact that HCMV is not yet included in the oncogenic viruses list, its possible contribution in carcinogenesis as initiator or promoter is significantly reported suggesting that HCMV and tumors express a symbiotic relationship (26–29). HCMV aids the tumor to escape immune surveillance by encoding viral proteins and inducing various cellular factors, in addition to the HCMV-induced immune tolerance which favors tumor growth. In return, HCMV harbors in the immunologically weak environment of the cancerous cells (6). This review accentuates the considerable influence of HCMV on the immune landscape and its oncomodulatory signals that might contribute to oncogenesis.

## Host Immune Responses Against HCMV Infection

HCMV, a double-stranded DNA (dsDNA) genome beta-herpesvirus is considered the largest virus among the human herpesviruses (30). Upon HCMV infection and despite the counteracting host response, this virus powerfully adapts to the human immune system. HCMV is certainly not eradicated from the HCMV-positive immunocompetent patient, in whom the virus establishes latency (31). Thus, the human immune system is incompetent to clear the latent HCMV, however it mounts an immune defense targeting multiple viral proteins (8). Due to the existing coevolution between HCMV and the host immune system for millions of years, it’s informative to study the immune defense strategies and pathogen counterstrategies (12). Innate immunity, in addition to adaptive humoral and cell-mediated immune responses, are induced by HCMV infection; such responses lead to the resolution of acute primary infection (5). Such immune responses differ during distinct life stages; throughout pregnancy, maternal anti-HCMV antibodies participate in preventing congenital fetal CMV infection (32). In addition, studies have shown that despite the detection of primary humoral and cellular immune responses in neonates, cell-mediated immune responses are delayed compared to adults which justifies the reason behind uncontrolled viremia and serious clinical harm in early life CMV infections (8, 32, 33). CMV-specific CD8^+^ T-cell responses in congenitally infected newborns were characterized by lower IFN-γ levels and elevated levels of IL-8 compared to adults (33). Finally, elderly people have increased sensitivity and susceptibility to serious infections and diseases most likely due to immunosenescence (14).

### HCMV Entry

HCMV exhibits a wide host cell range, possessing the ability to infect several cell types for instance endothelial cells, epithelial cells, fibroblasts, smooth muscle cells, leukocytes, and dendritic cells (DCs) (8, 34). In healthy persons, HCMV initiates its replication in the mucosal epithelium; thereafter, it disperses to monocytes and CD34+ cells, where it institutes a latent infection. Upon differentiation of HCMV-infected monocytes into macrophages, a viral infection could be initiated (35). Infection of both hematopoietic and endothelial cells systemically eases the viral spread within the host (36), unlike prevalent cell types infection including smooth muscle cells and fibroblasts which enhances efficient proliferation of the virus (35).

### Innate Immunity

As HCMV enters the cells, virions are firstly recognized by the host thus activating multiple pathways and strategies of innate immunity which is known as the primary host defense against HCMV infection (8). These involve inflammatory cytokines, type I interferon (IFN), and upregulation of CD80 and CD86 (37) that are essential for limiting pathogen’s spread and thereafter priming the adaptive immune response (5, 8). The stimulation of the NF-κB pathway and predominant inflammatory cytokines production for example interleukin-6 (IL-6) and tumor necrosis factor-alpha (TNF-α) (38) result from the interaction of viral envelope glycoproteins B (gB) and glycoprotein H (gH) with the immune-sensor molecules namely, toll-like receptors 2 (TLR2) (5, 12, 39, 40). Such inflammatory cytokines are capable of inducing and triggering phagocytic cells, such as dendritic cells, which have the ability to clear HCMV-infected cells (5, 8, 38). In the initial infection sites, NK cells are activated to eradicate HCMV-infected cells by the liberation of cytotoxic proteins (38). Furthermore, studies have shown NK cells’ role in inhibiting HCMV transmission in fibroblasts, epithelial, and endothelial cells and this through inducing IFN-β in target infected cells (41) and secretion of IFN-γ (42). NK cells, crucial guards of the immune system, produce a cytokine environment that triggers the consequential maturation of adaptive immune responses particularly T-cells (5, 8, 43).

### Adaptive Immunity

The adaptive immunity which contributes to the control of HCMV infection is among the strongest responses in which it fully engages humoral and cellular immune responses. Adaptive immunity is necessary to fundamentally manage HCMV primary infection, afterwards HCMV will enter into a latent state (44). The development of a sustained adaptive immune response is essential to preserve HCMV latency, avert acute viremia, and avert lytic infection which, in contrast, is frequent in patients on immunosuppressive therapies and immunocompromised individuals often leading to unrestricted replication and clinically severe HCMV morbidity and mortality (45).

#### Humoral Responses

Following a primary HCMV infection, the initiation of a robust immune response to control HCMV is essentially required. Many evidences supported the role of humoral immunity in limiting viral propagation and HCMV severity through antibody production targeting multiple CMV proteins, envelope glycoproteins, and genes (8, 32). The key target for antibody neutralization against HCMV is gB since it is related to cellular adhesion and invasion; besides, gH is considered the secondary target as it is involved in the fusion of the host cell membrane with the viral envelope (37). Other targets include the structural tegument proteins (pp65 and pp150) and non-structural proteins (IE1) (8, 32). A study shows that pregnant women, primarily infected with HCMV, having HCMV specific IgM antibodies and missing neutralizing IgG antibodies are at greater risk of transmitting HCMV to their fetus in contrast to seropositive mothers experiencing a recurrent infection (45). Thus, underlying the critical role of humoral immunity, especially HCMV IgG, in controlling HCMV infection and spread.

#### T-Cell Mediated Immune Responses

Due to the fact that the immune response stimulated by primary infection does not eliminate HCMV, HCMV-specific CD4^+^ T-cells, CD8^+^ T-cells, and gamma delta (γδ) T-cells**** have been considered as critical players in restricting viral replication in hosts acquiring persistent infections (38, 46, 47). With regard to CD8^+^ T-cells, the CD8^+^ HCMV-specific T-cell response is targeted toward HCMV proteins which are being expressed at different stages of viral replication (IE, early, early-late, and late) in addition to other proteins possessing various functions (capsid, tegument, glycoprotein, DNA-regulatory, and immune escape) (37). It is worth noting that the most immunodominant antigens to which HCMV-specific CD8^+^ T-cells react are addressed toward IE1 (UL123), IE2 (UL122), and pp65 (UL83) (37, 48). Even though the major histocompatibility complex (MHC) class I-restricted CD8^+^ T-cell immune response role in targeting HCMV is evidently marked, there exists a significant indication that CD4^+^ T-cells are as well fundamental in controlling HCMV infections (37). Further studies reveal the attainment of a cytolytic potential by pp65-specific CD4^+^ T-cells and gB-specific CD4^+^ CTL *in vivo* where CD4^+^ T-cells released granzyme B in reaction to glia presenting endogenous gB (49). The recruitment of HCMV-specific T-cells into the memory compartment is stimulated by the relatively prolonged viral replication of HCMV since T-cells are mandatory to limit HCMV viral replication and impede certain diseases (50). HCMV-specific T-cell responses inflate throughout life leading to a significant fraction of memory T-cells in healthy seropositive individuals (50–52). Hence, HCMV-seropositive immunocompetent people maintain lifetime protection despite the insufficient or minimal HCMV-specific T-cell responses. Cellular responses from CD4^+^ and CD8^+^ T-cells vary among individuals (50). HCMV-positive serostatus has been associated with CD8^+^ T-cell compartment expansion, reduced CD4:CD8 T-cell ratio as well as alterations in the expression of CD8+ T-cell senescence related markers (53, 54). These senescent cells are characterized by a progressive loss of CD28 and CD27, upregulation of CD57 expression which is known as the classical immune senescence marker, replicative senescence, and shortened telomeres resulting in a limited cell proliferation capacity and finally the inability to eliminate the HCMV infection (14, 55). Other cellular responses include γδ T-cells and NK cells. Although the previously mentioned cells are not targeted specifically against HCMV, they can still successfully lyse HCMV-infected endothelial cells and fibroblasts in consequence of a cellular stress response that upregulates the endothelial protein C receptor (EPCR) in addition to CD54 (Intercellular adhesion molecule-1, *ICAM-1*) (56). γδ T-cells contribute to dual immune response, anti-infectious and antitumor. Activated γδ T-cells are essential immune effectors against HCMV in which they stimulate IFN-γ and TNF-α production that may synergize to inhibit HCMV replication (38).

## HCMV Persistence Despite Antiviral Immunity

In healthy individuals, the operative homeostatic equilibrium established between HCMV and the host prevents serious HCMV complications. Conversely, in an immunocompromised host, fetus, and neonates, HCMV infection can cause multiple forms of clinical harm (8). Disequilibrium in immunocompromised patients can result in unhindered viral replication followed by the reactivation of the latent virus, with subsequent morbidity and mortality (5). Despite a powerful immunity involving both arms of the immune system, HCMV establishes latency. In that context, HCMV-encoded determinants of tropism for endothelial cells, an imperative objective of the infection, were considered. It was stated that in endothelial cells the UL133-UL138 locus, encoded in the ULb′ region of the HCMV genome, is essential for the viral late-stage response (57). In infected cells, this locus was mandatory for preserving membrane organization and is required for the progeny viruses’ maturation. However, it’s not necessary for early/late gene expression or viral genome synthesis. Viruses missing the *UL133-UL138* region, produce progeny viruses that are deprived of tegument and envelopes, leading to deficient viral yields. *UL135* and *UL136* genes, encoded in the UL133-UL138 region, promote viral maturation. Additional recent data propose that this locus involves the main molecular switch among latency and reactivation, including the opposing roles of *UL135* and *UL138*. Moreover, a study reported that the outcome of antiviral immunity might be influenced by numerous viral determinants, including HCMV strain, virulence, MHC I downregulation, and other escape strategies elicited by HCMV during the early virus-host interaction (3).

## HCMV Escape Machineries and Immunosuppression

**Figure 1:**
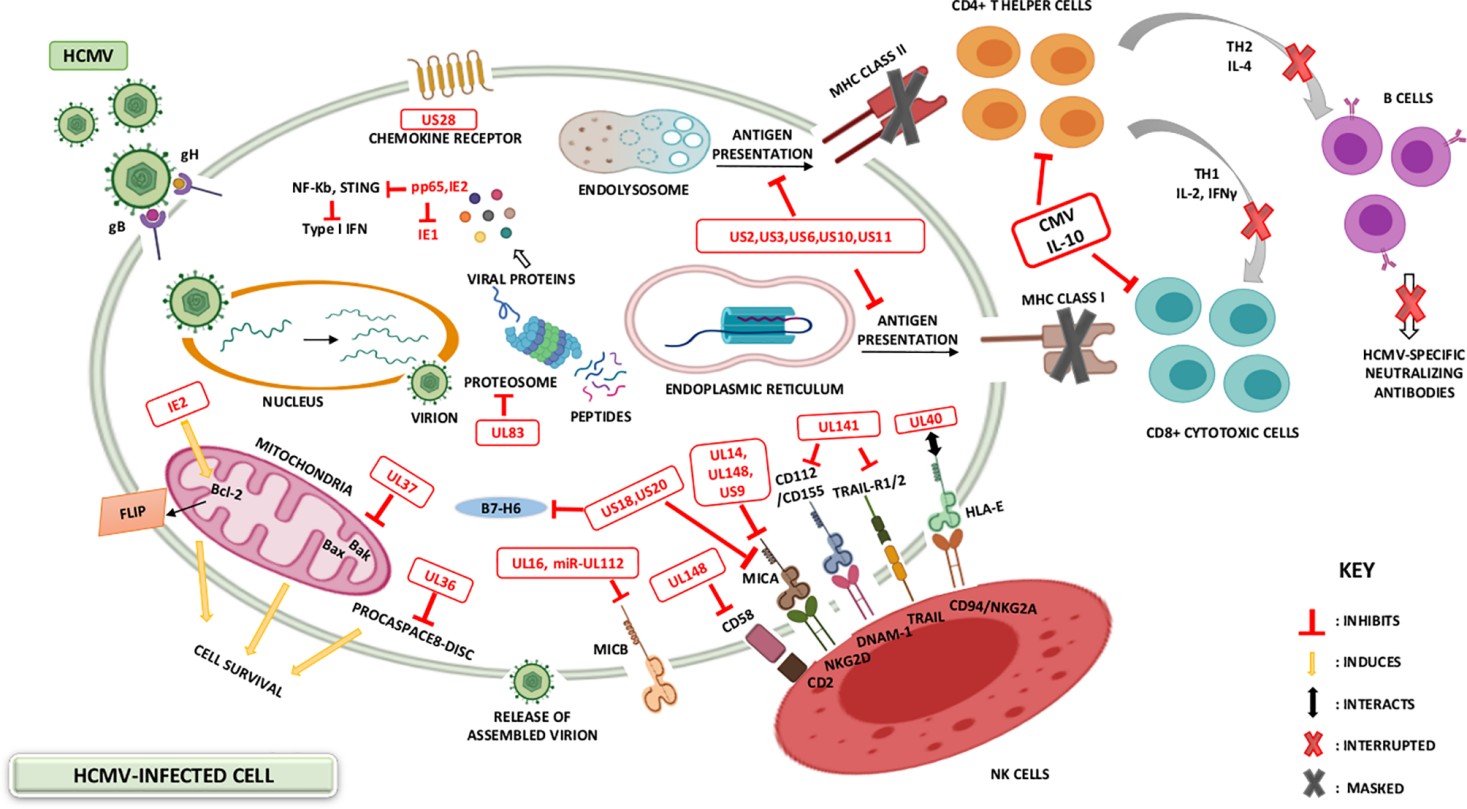
HCMV-induced modulation of the host immune system. The battle between the host immunity and HCMV is permanent, with HCMV developing various mechanisms to evade the host immune system. Immunosuppression may be ascribed to the variety of immune modulators encoded by HCMV-specific gene products. HCMV viral genes prevent MHC Class I and II antigen presentation and thus interfere with interferon responses, NK cell recognition as well as CD4^+^ and CD8^+^ T-cell recognition. Additional HCMV genes for instance IL-10 homologue (UL111a), and viral proteins acting as receptors for host inflammatory cytokines (US28), further suppress the host immune responses.

HCMV has evolved manifold immune evasion strategies that modulate the host immune system and promotes more efficient infection and dissemination within the host. A chief evasion strategy depends on hindering the MHC class I-restricted antigen presentation (58). Throughout the immediate early HCMV infection phase, a cytotoxic T-lymphocyte (CTL) response counteract antigenic peptides resulting from the IE1 transcription factor (59, 60). The matrix protein, pp65, possessing a kinase activity, phosphorylates the IE1 protein and specifically inhibit the presentation of IE-derived antigenic peptides to escape immune recognition of the early produced viral proteins (59). Knowing that pp65 is delivered directly into the cells during the viral fusion phase, HCMV will instantly escape from immunological surveillance, till further immune evasion related proteins are secreted (61). HCMV-specific viral proteins and genes that are associated with the host interferon responses (pp65), inhibit NK cell detection or activation (37, 62), and inhibit the recognition of CD4^+^ and CD8^+^ T-cell by preventing MHC Class I and II antigen processing and appearance (1, 37, 61). HCMV infected cells produce viral IL-10 homolog which further suppresses CD4^+^ and C8^+^ T-cell responses (1, 61). The previously mentioned evasion mechanisms are summarized in **Figure 1**.

In the absence of MHC class expression, HCMV must be susceptible to NK cell-mediated lysis; however, HCMV donates a large proportion of its genome to down-regulate the NK cell activity (63). Consequently, the surface expression of HLA-E and HLA-G is stimulated by gpUL40 and CMV-IL10 respectively (64–66). In addition, the expression of UL16 supports HCMV to block natural killer group 2D (NKG2D)-mediated NK-cell activation and this is by adopting a blocking strategy that hinders the binding of NKG2D to UL16 binding proteins (ULBPs) namely, ULBP1 and ULBP2, and to the MHC class I chain-related gene B (MICB gene) (37, 61). HCMV US18 and US20 proteins stimulate the deterioration of a major stress protein namely, MHC class I polypeptide-related sequence A (MICA); hence, preventing the NK cell from recognizing infected cells’ stress signals (67). Other machineries considered by HCMV to escape NK cell lysis involve the inhibition of NK cell-activating receptor (NKp30) by pp65 (37), UL122-encoded microRNA that represses *MICB* gene expression (68), and blocking of the expression of CD155 by HCMV-UL141 (37).

To counteract apoptosis and further evade the immune system, HCMV overexpresses anti-apoptotic proteins and inhibits pro-apoptotic molecules and death receptors. The former is achieved by upregulating B-cell lymphoma 2 (Bcl-2) in HCMV-infected cells (69) and expressing Fas-associated death-domain-like IL-1β-converting enzyme-inhibitory proteins (FLIP) by IE2 (70). On the other hand, pUL36 inhibits the induction of procaspase 8 to the death-inducing signaling complex (DISC) and pUL37 inhibits pro-apoptotic Bcl-2 members namely Bcl-2-associated X Protein (Bax) and Bcl-2 homologues antagonist/killer (Bak); thus, HCMV is hindering apoptosis through two distinct mechanisms (6, 61). Furthermore, HCMV has developed UL36 and UL37 proteins, which enhance the survival of infected cells; thus, stimulating viral dissemination within the host (37, 71). HCMV escapes complement attack by upregulating the host-encoded complement regulatory proteins (CRPs) (72) and by the ability of HCMV to integrate host cell-derived CRPs, CD55 and CD59 in its virions (6).

Lastly, HCMV produces the G-Protein-coupled receptors (GCRs) homologs US27, US28, UL33, and UL78 that might act as eliminators of chemotactic factors, thus hindering the inflammatory cells’ accumulation at the viral infection site (61, 73). These viral approaches secure novel viral progeny production and facilitate the spread to other hosts (61). US28 is usually expressed in the early infection phase; it shows the highest homogeneity to the CC chemokine receptor CCR1. It also binds the CC chemokines RANTES, monocyte chemoattractant protein-1 (MCP-1), monocyte chemotactic protein-3 (MCP3), macrophage inflammatory protein-1 alpha (MIP-1α), and macrophage inflammatory protein-1 beta (MIP-1β), in addition to the membrane-associated CX3C chemokine, fractalkine (37, 61, 74). US28 expression results in the stimulation of phospholipase C and NF-κB signaling. The US28-fractalkine interaction has been involved in cell targeting and viral dissemination (61). The transcription of *US28* takes place during productive and latent HCMV infection, which might justify the dissemination of latent HCMV (75, 76). Moreover, HCMV encodes a homolog of the immunosuppressive cytokine IL-10 (UL111a) (62, 71); it likewise possesses potent immunosuppressive traits, including the inhibition of mitogen-stimulated peripheral blood mononuclear cells (PBMCs) proliferation in addition to the blockade of pro-inflammatory cytokine synthesis in PBMCs and monocyte (37, 61). CMV IL-10 binds to the cellular human receptor of IL-10 despite its minimal homology to the endogenous cellular IL-10 (61). In addition, HCMV establishes immune tolerance by inducing the transcription and release of TGF-β which inhibits anti-viral IFN-γ and TNF-α cytokine production and cytotoxic effector activities of HCMV specific Th1 cells (77). Further homologs encoded by HCMV are UL144 which is a viral TNF receptor and an effective IL-8-like chemokine (viral CXC-1) prompting the chemotaxis of peripheral blood neutrophils (UL146) (37). Lastly, HCMV strategies that modify the cellular infected environment to restrict immune identification are known to be widely expressed during lytic infection; however, recent evidence shows that viral genes’ activity in preventing immune recognition is being remarkable even during latency phases (75). Recent data shows that the majority of the HCMV-encoded proteins and microRNAs (miRNAs) are expressed also during latent stages (75, 76) (**Table 1** and **Figure 1**).

**Table 1:** HCMV gene products involved in immunomodulation and their oncogenic characteristics.

HCMV Gene Products	Mechanism of Action	Possible Oncogenic Characteristic
***US2, US3, US6, US11***	➢MHC class I expression impairment, reducing HCMV antigen presentation toward CD8^+^ cells and evasion of CD8^+^ T-cell immune responses, superinfection (60, 78) ➢US2 down regulates MHC class II and reduces HCMV antigen presentation to CD4^+^ cells (79)	➢Preventing CD8^+^ mediated cytotoxic tumor killing (80)
***US18 and US20***	➢Interfere with B7-H6 surface expression including endosomal degradation, evades NK cells’ immune detection (81)	➢HCMV-immune evasion might indirectly affect tumor environment
***US28* (viral GPCR)**	➢Promotes chemotaxis (82, 83)	➢Cellular proliferation, tumor growth, enhanced angiogenesis and cell survival (84, 85)
***UL16***	➢Regulation of NK cell ligand NKG2D and impairing NK cells function (79)	➢Immune evasion, protects the cells from cytotoxic peptides-mediated lysis, and protects cancer cells from both NK and T-cells (80)
***UL40***	➢NK cell evasion (62) ➢HLA-E over expression (62), enhancing its interaction with the inhibitory receptor CD94/NKG2A (86)	➢HLA-E Over expression (1)
***UL83* (pp65)**	➢IE1 sequestration, repress proteasome processing, reduce NKp30 effect and delays antiviral gene expression (87)	➢Genomic mutation, immune evasion (84)
***UL122 (IE2)***	➢Overexpression of anti-apoptotic FLIP protein (60, 79)	➢Elevated immune suppression, cell proliferation, escaping growth suppressors and apoptosis (84)
***UL123 (IE1)***	➢Induction of TGF-β (82)	➢Cellular proliferation, genome instability and mutation, escaping growth suppressors, and ameliorated cell survival (84)
***UL82* (pp71)**	➢Inhibits antiviral response by binding to interferon stimulator gene (87, 88)	➢Cellular proliferation, escaping growth suppressors, and genomic mutation (84)
***UL111A (cmvIL-10)***	➢Inhibits MHC class II expression and suppresses CD4^+^ T-cell recognition (83, 89)	➢Immunosuppression, cellular proliferation, stimulates migration and metastasis, telomerase activation (84)
***UL142***	➢Inhibiting MICA (79, 90)	➢HCMV-immune evasion might indirectly affect tumor environment
***UL36***	➢Complexing with pro-caspase-8 thus suppressing its proteolytic stimulation and prompting its designation as viral inhibitor of caspase-8-induced apoptosis (vICA) (91, 92)	➢Enhanced cell survival
***UL37***	➢Inhibition of Bak and Bax protein, thus inhibiting apoptosis (79)	➢Enhanced cell survival
***UL76***	➢Activation of the DNA damage response thus inducing IL-8 expression (93)	➢Genome instability and mutation (84)
***UL97***	➢Forms a complex with pp65 and mediates immune evasion (5, 94)	➢Escaping growth suppressors (84)
***UL141- UL144***	➢Encodes for homolog of TNFR, hinders CD155 and CD112 expression (NK cell activating ligands) and the death receptor for the TNF family ligand TRAIL (5, 95)	➢HCMV-immune evasion might indirectly affect tumor environment
***UL145***	➢Depletion of helicase like transcription factor- (HLTF) through the recruitment of Cullin4/DDB ligase complex (96, 97)	➢Impeding innate immunity might indirectly affect tumor environment
***UL146***	➢Promotes neutrophil chemotaxis, immune escape (5, 98)	➢HCMV-immune evasion might indirectly affect tumor environment
***UL148***	➢CD58 Suppression; effective modulator of CTL function, amplify degranulation in cytotoxic T lymphocytes and NK cells against HCMV-infected cells (85)	➢HCMV-immune evasion might indirectly affect tumor environment
***miR-UL112***	➢Down regulation of MICB thus escaping NK cells, and decreased T-cell recognition (99, 100)	➢Exerts its oncogene function by directly targeting tumor suppressor candidate 3 (TUSC3) in GBM (101)
***LncRNA***	➢Function in both innate and adaptive immunity including the development, activation, and homeostasis of the immune system (102)	➢Cellular proliferation and transformation, facilitating signal transductions in cancer signaling pathways (84, 102), it’s also involved in angiogenesis (85)

## HCMV Complications Under Immunosuppression

The suppressive effects exerted by HCMV on the host immune system, HCMV persistence, dissemination, and reactivation result in dire consequences. The severe and mortal complications resulting from HCMV reactivation mainly occur in immunosuppressed and seriously ill immunocompetent individuals in whom the HCMV infection is accompanied by prolonged hospitalization and/or mortality. Furthermore, the manipulation of the host immunity can result in superinfections with other herpesviruses or bacteria, and exacerbate SARS-CoV-2 infections that are benefiting from the weakened immune system (18, 19, 103, 104). Since HCMV infection is known as a prevalent congenital viral infection, it might generate viral hepatitis with jaundice in addition to long-lasting disabilities, including hearing and visual damage, neurological impairments, and mental retardation (103). Additionally, studies show that HCMV has been detected in tissue specimens from immunocompetent individuals with inflammatory diseases, including atherosclerosis, psoriasis (6, 105), rheumatoid arthritis (24), inflammatory bowel disease (IBD) (105), and systemic lupus erythematosus (SLE); it has been concerned in the development of these diseases (103). HCMV leads to the development of restenosis after coronary angioplasty, chronic rejection of organ transplantation, chronic graft-*versus*-host disease in recipients of bone marrow transplants (24). Such observations infer the presence of an association between HCMV and autoimmune diseases. Knowing that the HCMV chemokine receptor homolog, US28, is considered a major co-receptor for several HIV strains, it provokes cell fusion with several forms of viral envelope proteins in addition to stimulating HIV-1 entry into HCMV- infected cells (106). Several epidemiologic studies suggested that HCMV infection has been linked to an elevated risk of cardiovascular death, one of which revealed that CMV seropositivity has been significantly associated with cardiovascular mortality (*P-value*=0.007) (107). This association was confirmed by another study showing an “increased six-year cardiovascular mortality” (*P-value*=0.021) (108). Further findings showed that CMV seropositive elderly presented elevated cardiovascular mortality compared to CMV-negative ones; the subhazard ratio for cardiovascular mortality was 1.95 (95% CI: 1.29–2.96, *P-value*=0.002) (109). The association between HCMV and vascular diseases is verified by the transient presence of US28 in smooth muscle cells which induces chemokinesis and chemotaxis (61). Moreover, since HCMV infection has been involved in producing modifications among the total T-cell population and adversely affecting to the immune well-being of elderly, it stimulates the occurrence of numerous age-related syndromes, and decreases efficacy of vaccines (3, 12, 110). Additionally, in elderly, HCMV could contribute to inflammation-mediated vascular pathology which is evaluated by determining systemic inflammation markers (C-reactive protein, IL-6, and TNF) and it might also cause direct vascular damage (2).

## HCMV Oncomodulation and Its Significance in Tumor Microenvironment

Alterations resulting from cancerous genetic and epigenetic instability provide recognizable antigens that are distinguished by the host immune system (111, 112). As cancer evolves, it can resist immune clearance by prompting tolerance in the presence of tumor-associated inflammatory cells (113). Consequently, a tumor microenvironment is generated and controlled by tumor-induced molecular and cellular interactions  (114) in which immune cells not only fail to exert anti-tumor effector functions, but also promote tumor development (113). Since CMV possesses different cellular signaling pathways, encodes many genes that exhibit immunosuppressive effects, and may empower cancer hallmarks, it thus plays an essential role in generating cancerous cells and has a fundamental impact on the tumor microenvironment (1, 29, 115).

Some studies put extra emphasis on the indirect role of CMV in cancer (115, 116). For instance, Dey et al. suggested that the association between glioma and CMV is an “observational association” (117). However, the prevalence of HCMV is remarkably high in several cancer forms (26, 118). Several research groups showed that over 90% of breast, colon, and prostate cancer, rhabdomyosarcoma, hepatocellular cancer, salivary gland tumors, neuroblastoma and brain tumors were positive for HCMV nucleic acids and/or proteins (26). HCMV DNA was confirmed in 100% of breast cancer and 91% of sentinel lymph nodes samples from the metastatic group (119). Moreover, a study conducted by Taher et al., showed HCMV detection in 98% of breast cancer derived metastatic brain tumors, suggesting a potential link between HCMV and metastatic cancer (120). HCMV was considered as a potential therapeutic target in metastatic cancer due to the expression of HCMV-IE protein in 53% of breast cancer samples which therefore resulted in shorter overall survival, and the detection of HCMV DNA and transcripts in 92% and 80% of the used specimens respectively (120). Another study showed the inversely proportional relation between HCMV-IE1 presence and hormone receptor expression suggesting HCMV role in hormone receptor-negative breast cancer tumors (121). HCMV IE1 and pp65 were present in 82% and 78% of colorectal cancer samples, and in 80% and 92% of adenocarcinomas, respectively. In colon cancer cells, these HCMV-specific proteins contribute to the induction of Bcl-2 and COX-2 proteins thus promoting colon cancer progression (122). Cobbs et al. showed that HCMV-IE1 was expressed in all studied glioma biopsy specimens, in all grades (II-IV) (123). Over again, HCMV-IE and late proteins were expressed in 100% and 92% of primary neuroblastoma samples respectively; notably, HCMV proteins were detected in CD133 and CD44-positive neuroblastoma cells (118). HCMV DNA was detected in the peripheral blood of GBM patients (80%), suggesting either HCMV reactivation or viral DNA shedding from HCMV-tumor cells (124). In addition, HCMV was detected in all evaluated preneoplastic and neoplastic prostate lesions (125). In Hodgkin’s disease cases, the HCMV infection frequency was 28.6% (126). Further, expression of HCMV was marked in the neoplastic epithelium of 97% of the carcinoma patients (127). 92% of the primary medulloblastoma cases expressed HCMV-IE protein while 73% expressed late viral proteins (128). Evident elevated survival rates were observed among HCMV positive glioblastoma patients who were on anti-viral therapy (valganciclovir) (120, 129). A 70% and 90% survival rate was proved with 6-month and continuous valganciclovir treatment, respectively (120). Despite the existing studies which describe the possible involvement of CMV in cancer, further large scale investigations are needed in addition to the necessity of novel epidemiologic studies knowing that the latter might be challenging to conduct especially among CMV-positive cancer patients.

HCMV infects multiple cell types including stem cells; referring to the fact that Thy-1 and platelet-derived growth factor receptor alpha (PDGFRα) stem cell markers enhance HCMV infection, stem cells are susceptible to HCMV infection (130, 131). Thus, stem cells serve as reservoirs for HCMV persistence and reactivation. The major stem cell regulator namely, Wnt tends to trigger HCMV transcriptional activation; hence, once HCMV disseminates to various body organs, viral expression occurs during patients’ lifetime in stem cells (132). It is worth noting that the latter increases the chance of accumulation of genetic mutations; thus, stem cells lose control over their self-growth and renewal, act as a cancer source, and become susceptible to oncogenesis in the presence of inflammation and altered DNA repair pathways (133). In return, HCMV can support stem cells survival which would potentially elevate oncogenesis. Studies reveal the effect of HCMV-IE1 protein in promoting the preservation of glioblastoma cancer stem cells through its induction of SRY-Box Transcription Factor 2 (SOX2), Nanog, Nestin, and octamer-binding transcription factor 4 (OCT3/4) where it’s considered as a key regulator of glioblastoma stem-like phenotype (134). In GBM cells, the induction of transcription factors that are crucial for cancer stem cell persistence, cancer growth, and signaling pathways associated with the epithelial to mesenchymal (EMT) phenotype are stimulated by HCMV IE1 expression (134, 135). Many studies proved that cancer stem cells infected with HCMV possess a progression potential in contrast to HCMV-negative cancer stem cells. Some HCMV strains, for instance HCMV-DB and HCMV-BL, are capable of transforming human mammary epithelial cells and producing a “transcriptional profile” associated with DNA hypomethylation that resulted in enhanced proliferation, activation of cancer stem cell, and EMT process (136, 137). Likewise, HCMV was proven to induce an EMT phenotype in colorectal carcinoma cells accompanied with amplified tumor proliferation and cancer cell invasion (136). In addition, IE1 expression was detected in CMV transformed HMECs (CTH) cells which as well express embryonic stem cell markers (138). HCMV IE1 and IE2 gene expression in addition to UL76 genes prompt DNA mutagenesis, chromosome breakage, and genomic instability. Such expression of HCMV gene products could affect the pathways of p53 and Rb tumor suppressors, and other pathways that are responsible for DNA repair (27, 139, 140). Presuming the role of HCMV gene products in causing DNA damage directly and indirectly, and stimulating proliferation in stem cells, HCMV may have the potential to initiate and promote tumor formation. The oncomodulatory potential of HCMV catalyzes an oncogenic process by producing viral proteins, helping tumor cells to evade the immune system, and preventing and/or delaying cell death. The lack of HCMV specific cellular immune responses in these immune-privileged tumor sites would definitely enhance HCMV replication. On the other hand, cancer cells on their own can escape immune responses by diverse mechanisms. Thus, the combination of intrinsic cellular with viral immune escape machineries in cancer cells may offer an environment which enhances HCMV replication and boost cancer cells to evade from immune surveillance showing the bidirectional relationship between tumor cells and HCMV (25, 141). It’s worth mentioning that HCMV can activate many of the tumor pathways’ hallmarks including uncontrolled inflammation, myeloid cells’ infiltration, immune modulation, angiogenesis, and metabolic reprogramming. Production of inflammatory cytokines including RANTES, MCP-1, MIP-1a, IFN-γ, TNF- α, IL-4, IL-18, and IL-8F is induced by HCMV (142, 143). The HCMV-US28 chemokine receptor strongly promotes the expression of the NF-kB, COX-2, IL-6, and p-STAT-3 which could initiate oncogenic pathways (144, 145). Upon HCMV infection of human cancer stem cells and in the presence of cmvIL-10, cancer stem cells can induce macrophage reprogramming “M2 phenotype” in the tumor microenvironment hence favoring the appearance of tumor-associated macrophages (TAMs) and enhancing other immunomodulatory, oncogenic, and angiogenic cytokines’ expression such as STAT3 and vascular endothelial growth factor (VEGF) (146–148). Similar to US28, the cmvIL-10 chemokine which is known to be expressed in latency phase and tumor cells can enhance cancer cell invasion (149). In addition, HCMV can guarantee neutrophils and mononuclear cells survival, which can support a quick oncogenesis *via* the activation of an angiogenic switch (150, 151). Further, long non-coding RNAs (lncRNAs) were described as efficient players not only in facilitating signal transductions in tumor signaling pathways but also in promoting tumor evasion from immunosurveillance. It has been also shown that immune cells for instance, T-cells, B-cells, dendritic cells, macrophages, and myeloid cells control tumor immune responses *via* lncRNAs linked pathways (152, 153). In CTH cells, HCMV lncRNA4.9 was formerly detected in tumors isolated from xenograft NSG mice injected with CTH cells, as well as in human breast cancer biopsies (137). Moreover, several studies specified that modifications in gut permeability and intestinal microbiota translocation can stimulate chronic inflammation as well as causing auto-immune and neoplastic diseases (154, 155). Knowing that CMV presence was associated with upregulation of various cytokines, elevated epithelial gut damage, microbial translocation, and systemic inflammation (156, 157), it might play a part in eliciting carcinogenesis. All in all, these studies indicate that HCMV can be actively involved in enabling cancer progression and this is through inducing certain pathways that give rise to epigenetic modifications, and promoting the activation of cancer stem cell, angiogenesis, invasion, and an EMT phenotype (136, 158, 159). Certainly, one of the limitations for assessing the effect of HCMV on immunity and cancer progression is that the majority of the investigations were done on the high risk CMV-positive subpopulation which might involve diverse immunomodulation sociodemographic and environmental co-factors other than HCMV status as well as divergent lifestyles and medical history. Therefore, prospective studies are highly required to rule out other immunomodulatory factors and precisely evaluate the impact of CMV on host immunity.

**Figure 2:**
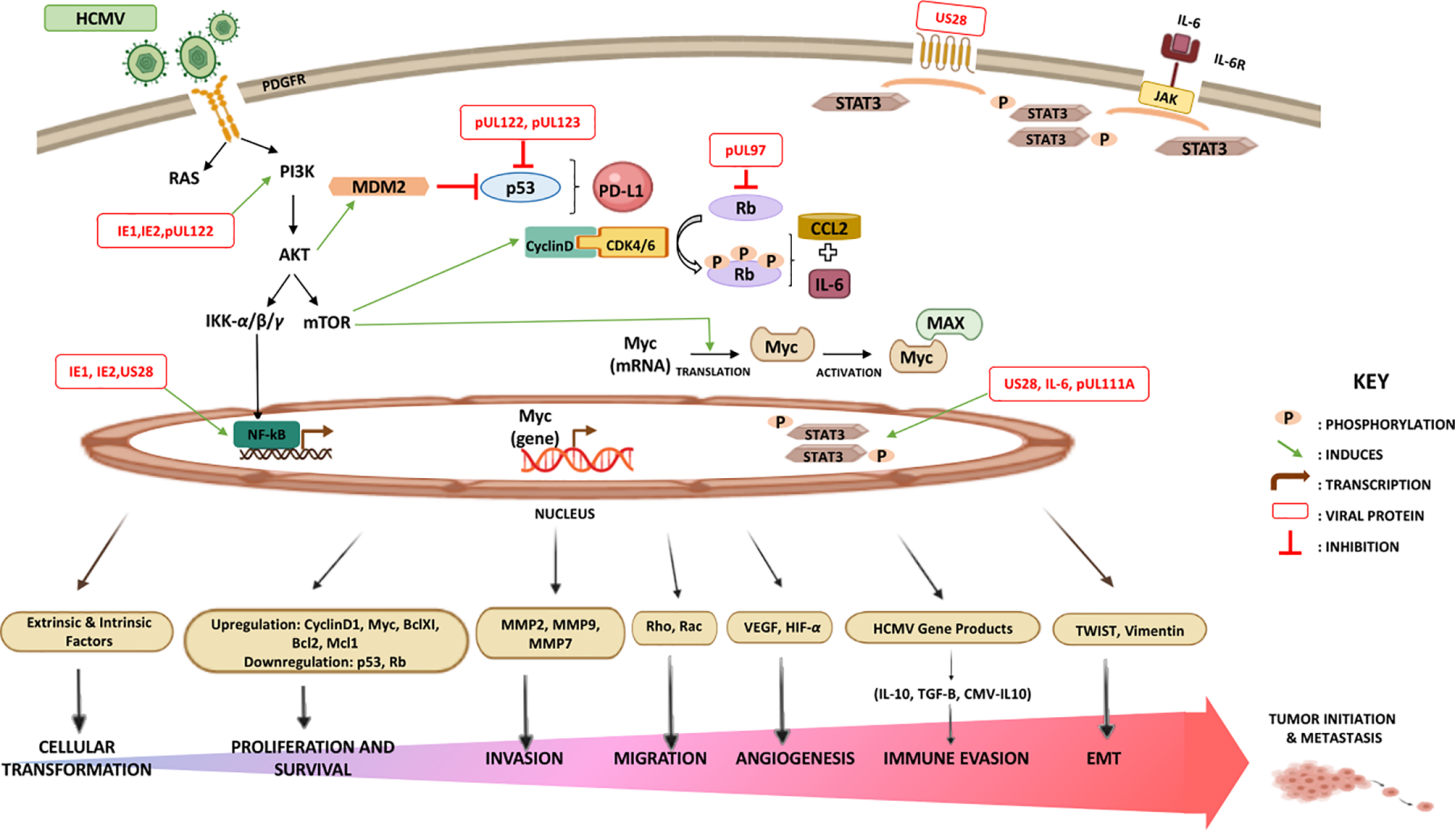
Major signaling pathways stimulated by HCMV that modulate the immune landscape. HCMV-infected cells produce elevated levels of interleukin-6 (IL-6) that activates signal transduction *via* IL-6 receptor (IL6R)-STAT3 axis. US28, an active chemokine receptor, also plays a major role in activating STAT3 in cancer cells. The combination of STAT3 activation and the impact of HCMV on cancer cell apoptosis, invasion, migration, adhesion, angiogenesis, and immunogenicity significantly exacerbates malignancy. In contrast to p53 and Rb, the upregulation of Akt, Myc, PD-L1, and CCL2 strongly exerts immunosuppressive and oncomodulatory effects. HCMV-induced alterations in the TME may contribute to oncomodulation.

Nonetheless, it is noteworthy that the oncogenic potency of HCMV clinical strains varies between low and high-risk strains. HCMV-DB and HCMV-BL have been classified as high-risk strains in which they possessed their oncogenic potentials in acutely infected human mammary epithelial cells (HMEC) *in vitro* showing sustained transformation. These high-risk strains were characterized by elevated *Myc* expression, PI3K/Akt pathway activation, and *p53* and *Rb* gene repression (138). With regard to immune responses, Myc suppresses immune surveillance by modulating the expression of the innate immune regulator (CD47, also known as integrin-associated protein) and the adaptive immune checkpoint namely, programmed death ligand 1 (PD-L1, also known as CD274 and B7-H1) (160–164). Further, Myc regulates thrombospondin-1 (161) and Type 1 IFN (165, 166). Hence, Myc initiates and maintains tumorigenesis through the modulation of immune regulatory molecules. PI3K/Akt activation induced inflammation and immunosuppression through nitric oxide synthase (NOS) overexpression; thus, resulting in tumor initiation *via* the activated Notch pathway leading to tumor progression (167). On the other hand, suppression or mutation of p53 has been shown to decrease MHC-I presentation, increase STAT3 phosphorylation, upregulate PD-L1 *via* microRNA (miR34), elevate pro-inflammatory chemokine and cytokine production, and indirectly upregulate the expression of chemokine receptors (CXCR4 and CXCR5) (160, 168, 169). Loss of Rb leads to the increase in CCL2 and IL6 secretion and this is because of the elevated fatty acid oxidation (FAO) activity and enhanced mitochondrial superoxide (MS) production (170). Indeed, those molecular alterations have been linked to immune suppression in the tumor microenvironment indicating that only high-risk HCMV strains possessing oncomodulatory properties are potentially involved in the oncogenesis process as described previously (84, 138) (**Figure 2**). In line with the previously presented epidemiological studies, and since HCMV was confined within tumors correlating positively to poor prognosis, as well as the potential of HCMV gene products in regulating tumorigenic pathways and processes linked to cancer hallmarks, and finally the HCMV broad tissue tropism, we infer that HCMV possesses distinctive mechanisms in cancer progression (26, 29, 171).

## Therapeutic Approaches in High-Risk Populations

The fact that HCMV is highly prevalent in different cancer forms, opens up the possibility to manage such cancers with anti-HCMV medications. Currently, two major approaches are being chased; the first emphases on antiviral therapy while the other targets HCMV directed immunotherapy. The core approach to antiviral therapy involves the use of valganciclovir. The rationale behind using valganciclovir is suppressing HCMV replication in HCMV-positive glioblastoma (GBM) cells leading to the repression of virus-mediated tumor-promoting strategies. Despite its viral replication suppression, valganciclovir doesn’t eradicate the virus. Thus, short-term valganciclovir treatment wouldn’t be ideal for treating glioma patients, necessitating long-term treatment to maintain the tumor suppressive properties (116). Interestingly, valganciclovir treatment outcome was improved in combination with celecoxib (COX-2 specific inhibitor). Since glioblastoma, medulloblastoma, and neuroblastoma tumors show high expression of cyclooxygenase-2 (COX-2) and nonsteroidal anti-inflammatory drugs (NSAIDs) levels, COX-2 and PGE2 inhibitors possess a profound effect on tumor growth. The two inhibitors competently block HCMV replication and limiting the US28-expressing tumor cell growth. Therefore, the significant effects behind the use of aspirin and other NSAIDs in tumor prevention investigations could be somewhat due to the suppression of HCMV replication in pre-malignant lesions (80). The existing link between CMV and cancer creates a new avenue for immunotherapeutic strategies that target CMV such as, adoptive T-cell transfer and vaccine approaches (172). During adoptive lymphocyte transfer (ALT), autologous T-cells are expanded and activated *ex vivo* against the tumor. After that, they are transferred into patients where lymphodepletion stimulates a substantial proliferation of T-cells and intensifies tumor-specific immunity (173, 174). There exist various ongoing clinical studies assessing the effectiveness of adoptive T-cell therapy using HCMV-specific T-cells, or DCs with CMV-pp65 RNA in order to vaccinate GBM patients. CMV-specific T-cells, especially pp65-specific T-cells, favorably infiltrate glioblastoma tumors and were able to stimulate glioblastoma cells’ killing (173). The fact that a high percentage of GMB samples were HCMV-positive has led to potential immunotherapy targets for GBM treatment. HCMV-specific proteins (IE1, pp65, and gB) are being investigated for the development of immunotherapy targets (116, 174). Interestingly, a study showed that CMV-stimulated NK cells and γδ-T-cells might have antineoplastic potential and CMV reactivation has been associated with minimal risk for relapsed leukemia in hematopoietic stem-cell transplantation (HSCT) patients (173). Since oncolytic virotherapy has been recognized as a promising approach for treating cancers in recent years, the use of “oncolytic CMV therapy” in combination with anti-tumor medications, immune checkpoint inhibitors (targeting CTLA4 and PD-L1), epigenetic therapeutics, or as “HCMV/HSV-1 oncolytic virus” could be regarded as one of the most intriguing antitumor approaches (175).

**Figure 3:**
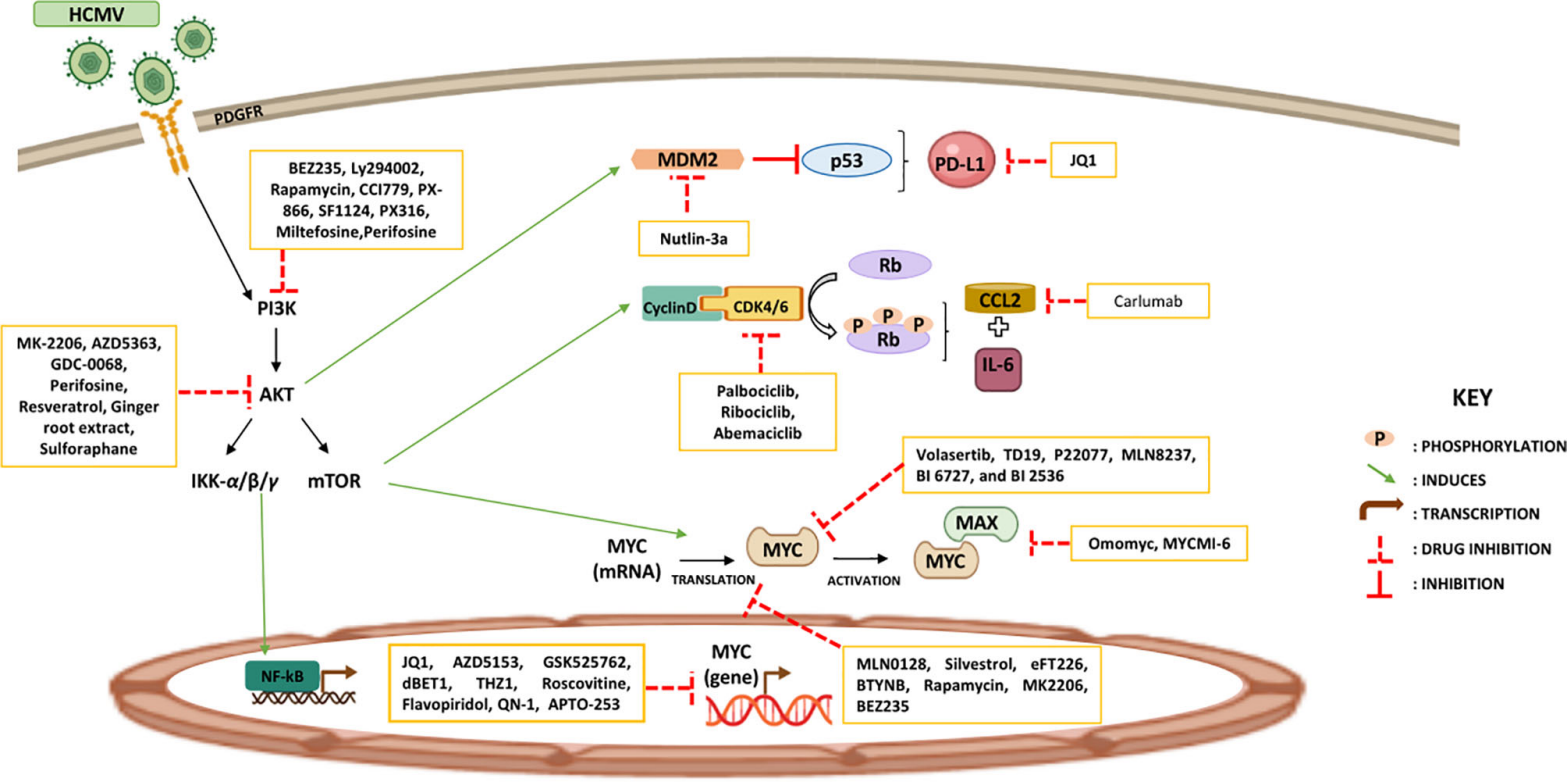
Potential therapeutic approaches reinstating tumor immunity. Certain therapeutic strategies are used to increase tumor suppressor proteins and reduce oncoproteins’ expression with the aim of restoring the immune response against tumors.

Major approaches used to target Myc are mostly targeting *Myc* gene transcription (JQ1, AZD5153, GSK525762, dBET1, THZ1, Roscovitine, Flavopiridol, QN-1, and APTO-253), inhibiting Myc mRNA translation (MLN0128, Silvestrol, eFT226, BTYNB, Rapamycin, MK2206, BEZ235), targeting Myc oncoprotein stability (Volasertib, TD19, P22077, MLN8237, BI 6727, and BI 2536), controlling Myc-Max interactions (Omomyc and MYCMI-6), and blocking Myc’s accessibility to other genomic targets (Sulfopin) (176–178). Further, bromodomain and extraterminal protein (BET) inhibitor, JQ1, decreased expression of PD-L1 and CD47 resulting in the recruitment of T-cells. Hence, drugs targeting Myc-associated pathways may be used to modify the expression of immune checkpoints (179). Furthermore, the PI3K/AKT pathway is activated in cancer; thus, identifying AKT inhibitors that can block PI3K/AKT signaling could attenuate tumor growth and recover immune responses. AKT inhibitors are classified in Synthetic (MK-2206, AZD5363, GDC-0068, Perifosine) and natural AKT inhibitors (Resveratrol or grape powder, Ginger root extract, Sulforaphane) (180, 181). Few drugs in clinical use or preclinical assessment have been verified to directly or indirectly target PI3K signaling such as BEZ235, Ly294002, Rapamycin, CCI779, PX-866, SF1124, PX316, Miltefosine, and Perifosine (178, 181). Reactivating tumor suppressors is a substantial pharmacological challenge; restoring p-53 activity stimulated innate immunity particularly DC activation, and it also promoted adaptive immunity. Nutlin-3a, mouse double minute 2 homolog (MDM2) inhibitor, induces local p53 activation in the TME resulting in MDM2-mediated tumor cell apoptosis even in the presence of a sustained Notch activity (168, 182, 183). Re-expression of p53 was stimulated by Tamoxifen injections causing massive apoptosis (179). T-cell responses were driven by using p53 vaccines (ALVAC-p53 and MVAp53) or synthetic long peptides of p53 (169). Moreover, the highly selective cyclin-dependent kinases 4/6 (CDK4/6) inhibitors (Palbociclib, Ribociclib, and Abemaciclib) were proved to avert RB phosphorylation thus regulating MHC presentation, IFN-γ response, and IL-6 signaling (184–186). Carlumab, a human IgG1 monoclonal antibody, inhibited CCL2 and it consequently showed promising effects in both solid tumors and metastatic resistant prostate cancer. A distinct approach to CCL2/CCR2 interference, was hindering CCR2 using MLN1202 in bone metastasis (187). It has been shown that CCL2 knockout prompted marked suppression of TAMs-associated inflammatory cytokines (188); in addition, CCL2-CCR2 blockade exhibited tumor-suppressive function by blocking inflammatory monocyte recruitment within the tumor (170) (**Figure 3**).

Additionally, because of HCMV’s ability to establish latency and reactivate, CMV vaccines are presently being developed for clinical use. To prevent HCMV infection in tumor-independent settings, the development of an effective HCMV vaccine has been investigated despite being a struggle for a couple of years. Few have already granted the approval to a phase III clinical trial thus possessing promising outcomes (189). The investigated anti-CMV vaccine types include the live-attenuated (phase 2), recombinant subunit (phase 2), virus vectored phase (1, 2), chimeric peptidic (phase 2), enveloped virus-like particles (phase 1), plasmid-based (phase 3), and mRNA (phase 2) vaccines (190). mRNA-1647, a CMV vaccine covering six mRNAs that encodes pentamer and gB protein, is designed for CMV prophylaxis; a phase 3 study will be initiated to assess the prevention of primary CMV infection in women of childbearing age (ClinicalTrials.gov Identifier: NCT04232280). Recent data showed that HCMV could perhaps induce transformation by enhancing the expression of viral genes (for example, *UL69* gene). The presence of *UL69* gene in CTH cell cultures and UL69 DNA in the majority of breast cancer biopsies indicates a potential significance of *UL69* as a target in developing HCMV-vaccine (191). The usage of HCMV vaccines for the treatment of cancer patients generally and GBM patients in specific might be of high therapeutic value especially that HCMV has been shown to express oncomodulatory functions. Letermovir, an FDA approved novel terminase inhibitor, is currently used for CMV prophylaxis as it selectively compromises CMV replication. It’s characterized by its high potency compared to ganciclovir and limited toxicity profile (192). There exists an ongoing phase 2 clinical trial that aim to assess the anti-inflammatory potential of letermovir in adults with HIV and asymptomatic CMV being on antiretroviral therapy-mediated suppression (ClinicalTrials.gov, Identifier: NCT04840199). Further investigations will be a welcome addition to evaluate the use of letermovir in averting CMV recurrence and treatment as well as to reverse letermovir resistance.

## Conclusion

Overall, HCMV-induced amplification of immune evasion mechanisms mediates oncomodulation and enables tumors to further escape immune surveillance and develop immune tolerance favoring other malignant phenotypes. HCMV, infecting many cell types, induces a pro-inflammatory environment and conquers specific immune responses thus creating an immunosuppressive TME. Nevertheless, getting to know viral immune evasion mechanisms will aid in understanding aspects of cellular as well as immunological function, and contribute to the enhancement of immunotherapies’ outcome and antiviral agents eliminating the virus from tumor tissues which could improve patient’s immune responses and suppress tumor progression. Taking into consideration the profound effects of HCMV on the quality of life, there remain further experimental studies to be performed in order to design effective interventions including vaccines or other approaches that reinforce immune homeostasis and maintain the adapted immune response to aging.

## Author Contributions

All authors listed have made a substantial, direct, and intellectual contribution to the work and approved it for publication.

## Funding

This work was supported by grants from the University of Franche-Comté, and the Région Franche-Comté (to GH). RB is a recipient of a doctoral scholarship from Hariri Foundation for Sustainable Human Development.

## Conflict of Interest

The authors declare that the research was conducted in the absence of any commercial or financial relationships that could be construed as a potential conflict of interest.

## Publisher’s Note

All claims expressed in this article are solely those of the authors and do not necessarily represent those of their affiliated organizations, or those of the publisher, the editors and the reviewers. Any product that may be evaluated in this article, or claim that may be made by its manufacturer, is not guaranteed or endorsed by the publisher.

## Glossary

ALT: Adoptive lymphocyte transfer
BAK: Bcl-2 homologues antagonist/killer
BAX: BCL2-Associated X Protein
Bcl-2: B-cell lymphoma 2
BET: Bromodomain and extraterminal protein
CDK: Cyclin-dependent kinases
COVID-19: Coronavirus disease 2019
COX-2: Cyclooxygenase-2
CRPs: Complement regulatory proteins
CTH: CMV transformed HMECs
CTLs: Cytotoxic T lymphocytes
DCs: Dendritic cells
DISC: Death-inducing signaling complex
dsDNA: Double-stranded DNA
EBV: Epstein-Barr virus
EMT: Epithelial to mesenchymal transition
EPCR: Endothelial protein C receptor
FAO: Fatty acid oxidation
FLIP: Fas-associated death-domain-like IL-1β-converting enzyme-inhibitory proteins
gB: Glycoproteins B
GBM: Glioblastoma
GCRs: G-Protein-coupled receptors
gH: Glycoprotein H
HCC: Hepatocellular carcinoma
HCMV: Human Cytomegalovirus
HHV-5: Human herpesvirus 5
HLTF: Helicase like transcription factor
HMEC: Human mammary epithelial cells
HSCT: Hematopoietic stem-cell transplantation
IBD: Inflammatory bowel disease
ICAM-1: Intercellular adhesion molecule-1
IFNs: Type I interferons
IL: Interleukin
IL6R: IL-6 receptor
JAK: Janus kinase
lncRNAs: Long non-coding RNAs
MCP-1: Monocyte chemoattractant protein-1
MCP3: Monocyte chemotactic protein-3
MDM2: Mouse double minute 2 homolog
MHC: Major histocompatibility complex
MICA: MHC Class I Polypeptide-Related Sequence A
MICB: MHC class I chain-related gene B
MIP-1α: Macrophage inflammatory protein-1 alpha
MIP-1β: Macrophage inflammatory protein-1 beta
miRNAs: microRNAs
MS: Mitochondrial superoxide
NK: Natural killer
NKG2D: Natural Killer Group 2D
NOS: Nitric oxide synthase
NSAIDs: Nonsteroidal anti-inflammatory drugs
Oct-4: Octamer-binding transcription factor 4
PBMCs: Peripheral blood mononuclear cells
PDGFRα: Platelet-derived growth factor receptor alpha
PD-L1: Programmed death-ligand 1
PI3K/AKT: Phosphatidylinositol-3-kinase and protein kinase B
SLE: Systemic lupus erythematosus
SOX2: SRY-Box Transcription Factor 2
STAT: Signal transducer and activator of transcription
TAMS: Tumor-associated macrophages
Th: T-helper
TLR2: Toll-like receptors 2
TME: Tumor microenvironment
TNFR: Tumor necrosis factor receptor
TNF-α: Tumor necrosis factor-alpha
TUSC3: Tumor suppressor candidate 3
UL: Unique long
ULBPs: UL16 binding proteins
US: Unique short
VEGF: Vascular endothelial growth factor
vICA: Viral inhibitor of caspase-8-induced apoptosis
γδ T-cells: Gamma-delta T-cells
